# Uncovering the mechanism of *Clostridium butyricum* CBX 2021 to improve pig health based on *in vivo* and *in vitro* studies

**DOI:** 10.3389/fmicb.2024.1394332

**Published:** 2024-06-14

**Authors:** Xin Liu, Xiaoyu Qiu, Yong Yang, Jing Wang, Qi Wang, Jingbo Liu, Jinxiu Huang, Feiyun Yang, Zuohua Liu, Renli Qi

**Affiliations:** ^1^National Center of Technology Innovation for Pigs, Chongqing, China; ^2^Chongqing Academy of Animal Science, Chongqing, China; ^3^College of Life Sciences, Southwest University of Science and Technology, Mianyang, Sichuan, China

**Keywords:** *Clostridium butyricum* CBX 2021, intestinal microbiota, intestinal health, weaned piglets, IPEC-J2 cells

## Abstract

**Introduction:**

As a symbiotic probiotic for the host, *Clostridium butyricum* (CB) has the potential to strengthen the body’s immune system and improve intestinal health. However, the probiotic mechanism of CB is not completely understood. The *Clostridium butyricum* CBX 2021 strain isolated by our team from a health pig independently exhibits strong butyric acid production ability and stress resistance. Therefore, this study comprehensively investigated the efficacy of CBX 2021 in pigs and its mechanism of improving pig health.

**Methods:**

In this study, we systematically revealed the probiotic effect and potential mechanism of the strain by using various methods such as microbiome, metabolites and transcriptome through animal experiments *in vivo* and cell experiments *in vitro*.

**Results:**

Our *in vivo* study showed that CBX 2021 improved growth indicators such as daily weight gain in weaned piglets and also reduced diarrhea rates. Meanwhile, CBX 2021 significantly increased immunoglobulin levels in piglets, reduced contents of inflammatory factors and improved the intestinal barrier. Subsequently, 16S rRNA sequencing showed that CBX 2021 treatment implanted more butyric acid-producing bacteria (such as *Faecalibacterium*) in piglets and reduced the number of potentially pathogenic bacteria (like *Rikenellaceae RC9_gut_group*). With significant changes in the microbial community, CBX 2021 improved tryptophan metabolism and several alkaloids synthesis in piglets. Further *in vitro* experiments showed that CBX 2021 adhesion directly promoted the proliferation of a porcine intestinal epithelial cell line (IPEC-J2). Moreover, transcriptome analysis revealed that bacterial adhesion increased the expression of intracellular G protein-coupled receptors, inhibited the Notch signaling pathway, and led to a decrease in intracellular pro-inflammatory molecules.

**Discussion:**

These results suggest that CBX 2021 may accelerate piglet growth by optimizing the intestinal microbiota, improving metabolic function and enhancing intestinal health.

## Graphical abstract

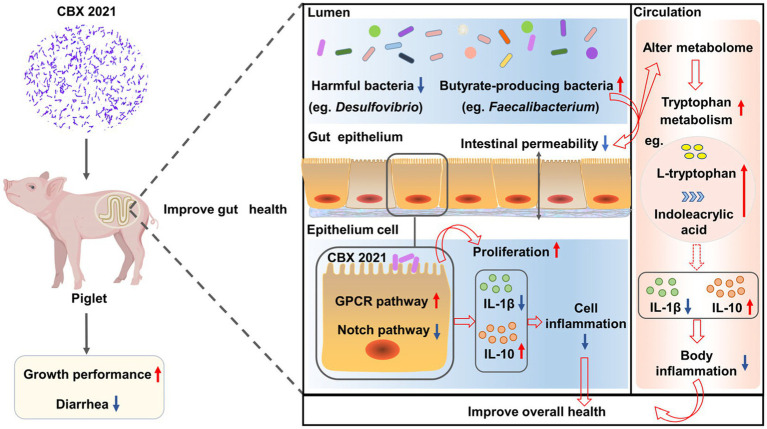

## Introduction

A healthy gastrointestinal tract is the cornerstone for improving animal growth performance and overall health. However, in modern intensive livestock production, weaned piglets experience drastic changes in diet, environment, and management that can disrupt normal GI function, resulting in delayed growth and compromised health (Tang X. P. et al., 2022). Multiple evidence suggests that probiotics can improve intestinal health in weaned piglets, thereby promoting body growth and overall health (Mun et al., 2021; Tang et al., 2021).

*Clostridium butyricum* (CB) serves as an effective probiotic for restoring the disrupted intestinal microbiota in livestock due to weaning stress. Additionally, CB has been proven to alleviate diarrhea and enhance growth performance (Lu et al., 2020; Ye et al., 2022). Extensive evidence shows that butyric acid, the major functional metabolite produced by CB bacteria, repairs intestinal mucosa damage, maintains intestinal immune homeostasis, and alleviates inflammation in the body (Abdelqader and Al-Fataftah, 2016; Chen G. X. et al., 2018). In recent years, CB has become popular in animal husbandry production as a probiotic preparation for its beneficial characteristics. Despite ongoing research, there is still much to uncover about the specific mechanisms through which CB regulates intestinal health and promotes growth.

Screening and confirming a suitable bacterial strain is crucial for the development of microbial ecological preparations due to the significant differences in physiological characteristics among the different strains from different sources. Our team isolated the *Clostridium butyricum* CBX 2021 strain from a healthy adult pig, which has shown a high capability for producing butyric acid. The strain was found to produce endospores and have a butyric acid production of 2.15 mg/mL during the exponential growth phase. Additionally, it is non-pathogenic to animals and can effectively restore antibiotic-induced intestinal dysbiosis and damage in mice by rebuilding the gut microbiota and optimizing metabolic function (Liu et al., 2023). Thus, it appears that CBX 2021 shows promise for application in the field of feed probiotics. However, the impact and mechanism of this strain on pig health, especially intestinal health, are currently unclear.

This study examines the impacts of the CBX 2021 strain on the growth and overall health of piglets. Various methods, including microbiome, metabolomics, and transcriptome analysis, were used in both *in vivo* and *in vitro* experiments to systematically reveal the potential mechanisms of its probiotic effects. The study provides guidance for developing and using new micro-ecological products.

## Materials and methods

### Bacteria and its preparation

*Clostridium butyricum* CBX 2021 strain was isolated and screened from the intestines of a healthy 6-month-old boar in our laboratory, and is currently stored at the *Guangdong Microbial Culture Collection Center* (GDMCC No.62503).

The CBX 2021 strain was incubated under anaerobic environment at 37°C for 12 h in reinforced *Clostridium* medium. For animal administration, the harvested bacterial solution (3 × 10^8^ CFU/mL) obtained at this time point was orally administered to weaned piglets. For cell culture assays, the bacterial solution was centrifuged at 5,000 × *g* at 4°C for 10 min to collect the bacteria. Then, the bacteria were resuspended in DMEM/F12 cell culture medium without penicillin/streptomycin (1.5 × 10^8^ CFU/mL) for cell experiments.

### Animal experiment design and sample collection

The experiment included forty-eight 28-day-old healthy piglets (8.16 ± 0.77 kg) split evenly into 2 groups, with 6 replicates per group and 4 piglets per replicate. The control (CON) group piglets were administered a 5 mL sterile saline orally, while the CB group piglets were given a 5 mL CBX 2021 bacterial solution (with an effective bacterial count of 3 × 10^8^ CFU/mL) orally once daily. The experiment lasted for 28 days and utilized a commercially available, standard-formulated feed. The composition and nutritional levels of it are displayed in Supplementary Table S1.

After the experiment concluded, one piglet from each replicate was chosen at random to gather fresh fecal samples for 16S rRNA sequencing analysis. Meanwhile, the venous blood of piglets was collected. After the blood samples stood overnight, the serum was collected by centrifugation at 3,000 × *g* at 4°C for a duration of 15 min and stored at −80°C for subsequent analysis of immune, growth-related hormones, and intestinal permeability.

### Growth performance

All piglets were weighed on an empty stomach on d 0 and 28, and the daily feed intake for each replicate was noted daily. These values are utilized in the computation of the average daily gain (ADG), average daily feed intake (ADFI), and feed-to-gain ratio (F/G).

Monitor the fecal consistency of each piglet during the trial, and document the number of piglets with diarrhea. Assess the piglet feces according to the following scoring standards reported in previous studies and calculate the frequency and index of diarrhea (Hung et al., 2019).

$$\begin{array}{l} \mathrm{Diarrhea}\;\mathrm{frequency}\;\left(\%\right)=\\ \;[\sum (\mathrm{number}\;\mathrm{of}\;\mathrm{piglets}\;\mathrm{with}\;\mathrm{diarrhea}\times \\ \;\mathrm{number}\;\mathrm{of}\;\mathrm{days}\;\mathrm{with}\;\mathrm{diarrhea})\;/\;(\mathrm{total}\;\mathrm{number}\\ \;\mathrm{of}\;\mathrm{piglets}\;\times \mathrm{test}\;\mathrm{days})]\times 100. \end{array}$$

$$\begin{array}{l} \mathrm{Diarrhea}\;\mathrm{index}=\mathrm{sum}\;\mathrm{of}\;\mathrm{diarrhea}\;\mathrm{scores}/\\ \;\left(\mathrm{total}\;\mathrm{number}\;\mathrm{of}\;\mathrm{piglets}\times \mathrm{test}\;\mathrm{days}\right)\text{.} \end{array}$$

### Blood biochemical indicators

As per the manufacturer’s guidelines, the ELISA kit (Enzyme-linked Biotechnology Co., Ltd., Shanghai, China) was utilized to measure serum immune indicators: immunoglobulin A (IgA), IgG, and IgM, as well as interleukin-1β (IL-1β), IL-10, and tumor necrosis factor-α (TNF-α); growth-related hormone indicators: growth hormone (GH), gastrin (GAS), ghrelin (GHRL), peptide YY (PYY), and glucagon-like peptide-1 (GLP-1); intestinal permeability indicators: D-lactic acid (DLA) and diamine oxidase (DAO).

### Fecal microbiome analysis

As previously mentioned, we extracted and purified total genomic DNA from piglet fecal samples (Wang J. L. et al., 2016). The quantitative PCR products were then examined through the Illumina NovaSeq platform for sequencing at Beijing Biocloud Biotechnology Co., Ltd. (Beijing, China^1^) via paired-end sequencing. The raw data can be found in the NCBI Sequence Read Archive (SRA) database and accessed using the login number PRJNA947735.

Sequencing reads were denoised using the DADA2 method and clustered into amplicon sequence variants (ASVs). The SILVA 138 database was used as a reference for classifying and annotating each representative sequence of the ASVs using a naive Bayes classifier, with a 70% confidence threshold. The unweighted unifrac distance algorithm was used for principal coordinate analysis (PCoA) at the bacterial genus level. ANOSIM function was used for statistical analysis. Student’s *t*-test or Mann–Whitney *U* test was used to analyze the differences in microbial distribution at phylum and genus level. Additionally, Linear discriminant analysis (LDA) effect size (LEfSe) was employed to identify biomarkers (from family to genus level), with a significance threshold set at an LDA value of ≥3.0.

### Serum metabolomics analysis

The serum samples from the piglets were sent to Beijing Biocloud Biotechnology Co., Ltd. (Beijing, China) for metabolite extraction, detection and analysis. The Progenesis QI software was utilized to process the raw data, including peak detection, calibration, and other essential operations for data processing. Upon peak area normalization, the processed data were exported for further analysis. The orthogonal partial least squares discriminant analysis (OPLS-DA) was utilized to compare and identify the overall metabolic variations in the CON and CB groups. Differential metabolites were chosen based on VIP values >1 in the OPLS-DA model and *p-*value <0.01. The pathway enrichment analysis of the differential metabolites was then conducted using the Kyoto Encyclopedia of Genes and Genomes (KEGG) database. The Spearman correlation algorithm was applied to analyze the relationships among the top 20 bacterial genera, differential metabolites, and serum biochemical indicators, generating correlation matrices to assess their associations.

### Cell and cell culture

IPEC-J2 cells were graciously provided by Dr. Yongjiang Wu (Chongqing University of Arts and Sciences, Chongqing, China). The cells were grown in DMEM/F12 medium supplemented with 10% fetal bovine serum (Gibco, Paisley, USA), 1% penicillin/streptomycin (Solarbio, Beijing, China), and 1% insulin-transferrin-selenium (Sigma-Aldrich, MO, USA). And they were incubated in biochemical incubators (5% CO_2_, 37°C).

### Determination of the adhesion ability of the CBX 2021 strain to IPEC-J2 cells

IPEC-J2 cells were inoculated in a 6-well plate (5 × 10^5^ cells/ well), and divided into 2 groups after the confluence reached 70–80%. CB group was exposed to CBX 2021 bacterial suspension (1.5 × 10^8^ CFU/mL, 2 ml/well), and CON group was treated with an equivalent volume of DMEM/F12 medium, with three replicates in each group. After incubation at 37°C for 4, 6, and 8 h, the cells were treated according to the standard procedure. Subsequently, they underwent fixation with 4% neutral formaldehyde for 15 min before being subjected to gram staining. The samples were examined under inverted fluorescence microscope with magnification of ×40 for analysis. Lastly, 25 cells were randomly selected, and the average number of CBX 2021 bacterial adhesion on visible cell surfaces was calculated and documented through photography.

### IPEC-J2 cell proliferation assay

IPEC-J2 cells were inoculated in a 96-well plate (1 × 10^4^ cells/ well) and grew to the above degree. The cells were classified into 3 distinct groups: the blank group (without cells), CON group, and CB group. Each group had 3 replicate wells (100 μl/ well). After the cells were incubated for 2, 4, 6, 8, and 12 h, the old medium was aspirated and DMEM/F12 medium with 10% CCK-8 was added (100 μl/ well) within the specified culture time, followed by 2 h of additional incubation. Then, OD values at 450 nm were determined using plate reader, and the cell proliferation activity was calculated at each culture time point to ascertain the optimal time for coculture of the bacterial strain and cells.


$$\begin{array}{l} \mathrm{Cell}\;\mathrm{proliferation}\;\mathrm{activity}\;\left(\%\right)=\\ \;\left[OD\;\left(CB\;\mathrm{group}\right)-OD\;\left(\mathrm{blank}\;\mathrm{group}\right)\right]/\\ \;\left[OD\;\left(\mathrm{CON}\;\mathrm{group}\right)-OD\;\left(\mathrm{blank}\;\mathrm{group}\right)\right]\times 100 \end{array}$$

### Determination of inflammatory factor secretion in IPEC-J2 cells

Cell processing and grouping were as described in the above adhesion experiment. After incubation for 6 h, the coculture supernatant was gathered to measure the secretion levels of IL-1β and IL-10 using porcine ELISA kits as per the manufacturer’s guidelines.

### RNA-seq and gene expression analysis

Cell processing and grouping were as described in the above adhesion experiment, and each group had 5 replicates. After incubation for 6 h, cell digestion and collection were performed using 0.25% pancreatase-EDTA (Sigma-Aldrich, MO, USA). The cell samples were then sent to Hangzhou Lianchuan Biotechnology Co., Ltd. (Hangzhou, China) for total RNA isolation, quality control, cDNA library construction, and transcriptome sequencing. The raw data can be found in the NCBI SRA database and accessed using the login number PRJNA948058.

The raw sequencing data underwent quality control, which involved removing reads that had low-quality bases and adapter contamination. Fragment per kilobase per million mapped reads were used to determine gene expression levels. DESeq2 was applied for the differential expression analysis, with the false discovery rate (FDR) controlled through the Benjamini-Hochberg procedure. The screening condition of differentially expressed genes (DEGs) was FDR < 0.05 and |log2(Fold change)| > 1. Additionally, the enrichment analyses were conducted using the OmicStudio cloud platform, including gene ontology (GO) term, KEGG pathway, and gene set enrichment analysis (GSEA). GSEA analysis relied on the GO database and involved calculating a normalized enrichment score (NES) for each gene set through 1,000 permutations of the genome. Gene sets with |NES| > 1 and FDR < 0.05 were considered significantly enriched in the GSEA analysis.

### Quantitative real-time PCR

The previously mentioned RNA samples underwent reverse transcription to generate cDNA, utilizing a commercially available reverse transcription kit (Yeasen, Shanghai, China) in a two-step reaction. Then, we followed the instructions provided by the manufacturer of the fluorescent quantitative PCR assay kit (Yeasen, Shanghai, China) in order to conduct our experiments. The 10 μl reaction mixture was prepared containing 1 μl of cDNA, 0.2 μl of upstream and downstream primer, 3.6 μl of RNA-free water, and 5 μl of SYBR fluorescent dye. The mixture underwent cDNA amplification and fluorescence signal collection using a real-time fluorescence quantitative PCR instrument (Thermo Scientific, Wilmington, DE, USA). The amplification program was performed as instructed. In this study, the PCR amplification efficiency was always between 95 and 100%, so the 2^-∆∆CT^ method was used to analyze the relative mRNA expression levels of the target genes mRNA (Livak and Schmittgen, 2001). A single internal reference gene GAPDH was selected for normalization based on previous studies (Wang H. et al., 2019; Wu et al., 2021; Li et al., 2023). Primers utilized in this research were synthesized by Shanghai Sangon Biotech Co., Ltd. (Shanghai, China) (Supplementary Table S2).

### Statistical analysis

After the data were preliminarily organization in Excel 2021, SPSS 26.0 software was used for statistical analysis. The Shapiro–Wilk test is used to evaluate whether the data obeys normality. Student’s *t*-test was conducted for the data that followed normal distribution and showed homogeneity of variance, while Mann–Whitney *U* test was used for non-parametric analysis when variance was unequal. The results are expressed as means ± SEM. Statistical significance was defined as *p* < 0.05 (*), *p* < 0.01 (**), and *p* < 0.001 (***). A significant trend was noted when 0.05 ≥ *p* > 0.10. Data visualization was carried out using GraphPad Prism 9.0 software.

## Results

### Supplementation with CBX 2021 improved the growth performance in weaned piglets

Table 1 presents the growth performance results of piglets. The CB piglets showed a 10.10% increase in body weight after 28 days compared to the CON piglets (*p* = 0.06). The ADG of CB treated pigs significantly increased by 24.95%, and F/G obviously reduced by 15.82% (*p* < 0.05). However, no difference in ADFI was witnessed between CON and CB piglets (*p* > 0.05). Compared to the CON group, CB piglets experienced a significant reduction in diarrhea frequency and diarrhea index by 36.15 and 40.74%, respectively (*p* < 0.05). The findings indicate that CBX 2021 improves the growth performance of piglets and helps manage post-weaning diarrhea.

**Table 1:** Effect of *Clostridium butyricum* CBX 2021 on growth performance in weaned piglets.

Items	CON group	CB group	*P*-value
Initial body weight (kg)	8.18 ± 0.22	8.14 ± 0.27	0.920
Body weight at d28 (kg)	17.62 ± 0.72	19.40 ± 0.54	0.064
ADG (g/d)	334.53 ± 26.53	418.00 ± 16.94	0.028
ADFI (g/d)	519.84 ± 29.47	553.69 ± 17.15	0.344
F/G	1.58 ± 0.09	1.33 ± 0.05	0.030
Diarrhea frequency (%)	37.48 ± 2.28	23.93 ± 3.14	0.006
Diarrhea index	0.81 ± 0.05	0.48 ± 0.08	0.005

*N* = 6.

Research suggests that there is a strong correlation between peripheral hormone secretion and improved growth performance (Li et al., 2021). Thus, we analyzed the serum levels of growth- and appetite-related hormones in piglets (Table 2). Compared with CON piglets, the of GH and GAS levels in the CB piglets increased by 6.33 and 7.74%, respectively (*p* < 0.05). The level of GHRL, a key appetite-stimulating hormone, showed a trend of increase by 3.90% (*p* = 0.06). Elevated levels of GAS and GHRL expression play a significant role in stimulating digestive fluid secretion, which is vital for increasing feed intake and conversion (Mazzoni et al., 2022; Rodríguez-Viera et al., 2022). However, there were no notable variations in the concentrations of the anorectic hormones PYY and GLP-1 between the piglets in the two groups (*p* > 0.05). The results indicate that CBX 2021 may positively impact the growth and development of piglets by enhancing endocrine hormone levels.

**Table 2:** Effect of *Clostridium butyricum* CBX 2021 on blood biochemical indicators in weaned piglets.

Items	CON group	CB group	*P*-value
Growth hormone (GH, ng/mL)	17.69 ± 0.32	18.81 ± 0.41	0.047
Gastrin (GAS, pg./mL)	628.07 ± 15.86	676.67 ± 13.98	0.037
Ghrelin (GHRL, pg./mL)	7083.87 ± 83.48	7360.06 ± 108.52	0.063
Peptide YY (PYY, pmol/mL)	15.91 ± 0.33	15.46 ± 0.38	0.384
Glucagon-like peptide-1 (GLP-1, pmol/mL)	12.11 ± 0.19	11.40 ± 0.38	0.119
Immunoglobulin A (IgA, μg/mL)	767.25 ± 11.41	812.55 ± 6.83	0.004
IgG (mg/mL)	24.68 ± 0.41	27.40 ± 0.59	0.002
IgM (mg/mL)	17.38 ± 0.47	18.65 ± 0.24	0.029
Interleukin-1β (IL-1β, pg./mL)	462.49 ± 14.96	390.66 ± 16.18	0.006
IL-10 (pg/mL)	121.55 ± 4.35	137.41 ± 5.06	0.032
TNF-α (pg/mL)	162.44 ± 3.16	165.97 ± 2.49	0.394
D-lactic acid (DLA, μmol/L)	347.52 ± 19.81	294.45 ± 7.71	0.034
Diamine oxidase (DAO, ng/mL)	227.45 ± 5.88	204.74 ± 5.56	0.014

*N* = 6.

### Supplementation with CBX2021 enhanced the immunity and intestinal barrier function in weaned piglets

The serum immunoglobulin levels in Table 2 reflect the humoral immune response level. The CB group piglets showed a significant increase of 5.90, 11.02, and 7.31% in IgA, IgG, and IgM levels, respectively, compared with CON piglets (*p* < 0.05).

Furthermore, we assessed serum levels of inflammatory mediators as indicators of weaning stress-induced inflammation within the body. The study revealed that compared with CON piglets, IL-10 content of CB piglets increased by 13.05%, and IL-1β content decreased by 15.53% (*p* < 0.05). However, there was no significant difference in TNF-α level between the two groups (*p* > 0.05).

The impact of CBX 2021 on piglet’ intestinal permeability was studied by measuring serum DLA levels and DAO activity. Elevated levels of DLA and DAO are typically indicative of heightened intestinal permeability (Cai et al., 2019). The findings indicated that the DLA level and DAO activity in the CB piglets were significantly reduced by 15.27 and 9.98%, respectively (*p* < 0.05) compared with CON piglets.

To sum up, the results indicate that CBX 2021 effectively enhanced immune response, strengthened intestinal barrier, and alleviated inflammatory stress in weaned piglets. This may lead to an overall improvement in the animals’ health status.

### Supplementation with CBX 2021 optimized the fecal microbiota in weaned piglets

**Figure 1:**
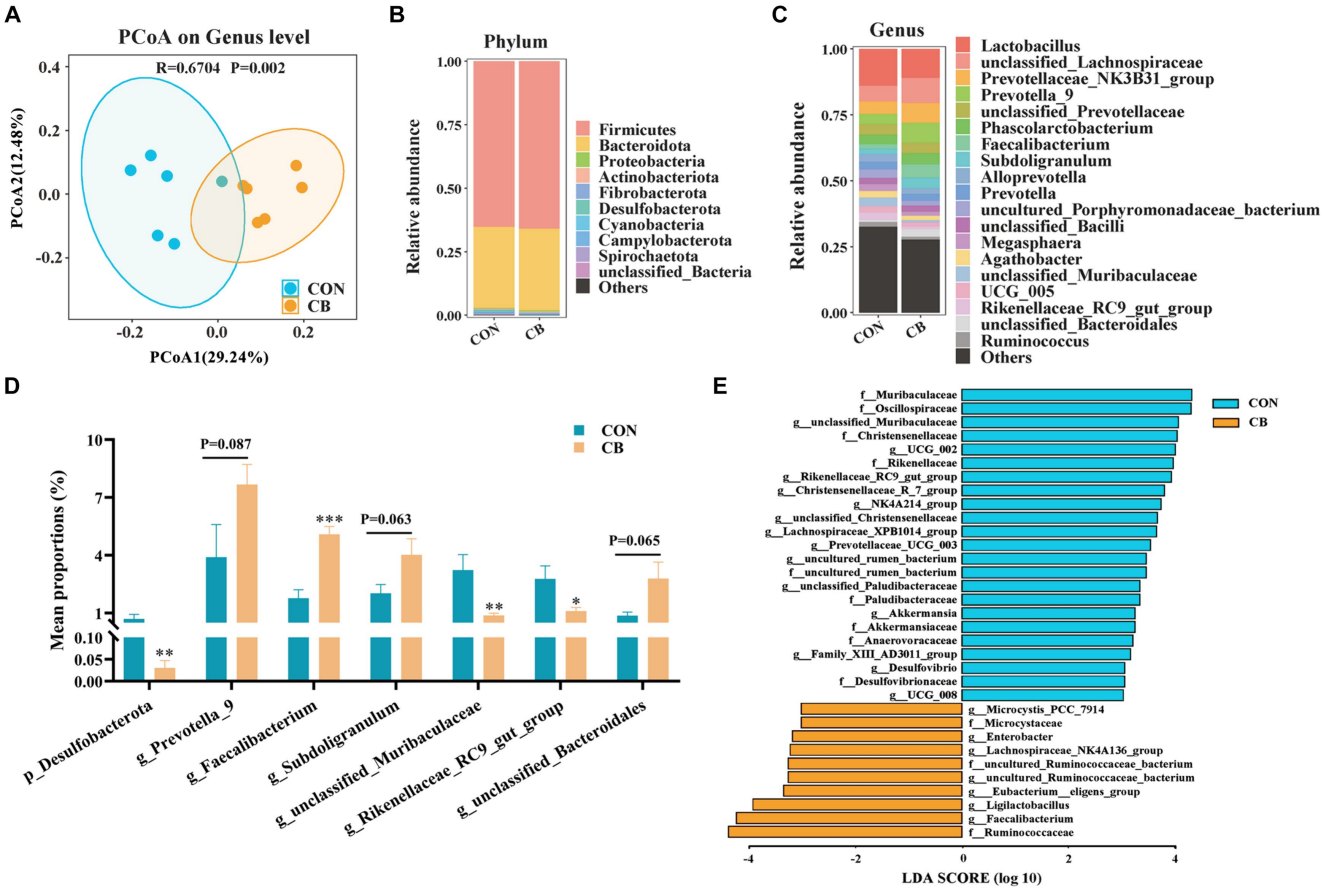
Effects of *Clostridium butyricum* CBX 2021 on fecal microbiota in weaned piglets (*n* = 6). **(A)** Principal coordinate analysis (PCoA) scatter diagram based on genus level. Column chart of species composition at the phylum **(B)** and genus **(C)** level. **(D)** Histogram of bacterial differences at the phylum and genus level. **(E)** Linear discriminant analysis (LDA) histogram of differential microbial community in the CON and CB group. Data are displayed as the mean ± SEM. *** Indicates *p* < 0.001, ** indicates *p* < 0.01, * indicates *p* < 0.05, and 0.05 ≥ *p* > 0.10 indicates a significant trend in comparison with the CON piglets.

A balanced microbial community structure contributes to immune system development and intestinal health (Wang et al., 2018; Wu et al., 2023). Here, we conducted an analysis on the diversity and structure of fecal microbiota by 16S rDNA sequencing to evaluate the gut bacterial community of piglets. The PCoA scatter plot, which illustrates the β diversity of microbiota, showing that the microbial distribution of the two groups of piglets is obviously different (*R* = 0.670, *p* = 0.002, Figure 1A). This result indicated that supplementation with CBX 2021 obviously altered the composition of microbial community in piglet feces.

Subsequently, an analysis of the bacterial population structure was conducted, focusing specifically on phylum and genus levels (Figures 1B,C). A combined total of 12 bacterial phyla were recognized in both groups of piglets. *Firmicutes* and *Bacteroidota* were the main phyla among them, comprising over 97% of the total abundance (Figure 1B). Notably, the abundance of *Desulfobacterota* was significantly decreased by CBX 2021 treatment (*p* < 0.05, Figure 1D). Figure 1C illustrates the relative abundance of the top 20 genera. *Faecalibacterium*, a butyrate producer, had a higher relative abundance in piglets receiving CBX 2021 intervention than CON piglets (*p* < 0.05). Additionally, there was an increasing trend in the number of helpful bacteria, particularly *Prevotella_9* (*p* = 0.087), *Subdoligranulum* (*p* = 0.063), and *unclassified_Bacteroidales* (*p* = 0.055), with their numbers 1.97-, 1.99-, and 4.65-fold higher than those of the CON piglets, respectively (Figure 1D). Furthermore, the addition of CBX 2021 caused a substantial drop in the number of proinflammatory bacteria, including *unclassified_Muribaculaceae* and *Rikenellaceae_RC9_gut_group* (*p* < 0.05, Figure 1D). The findings suggest that exogenous administration of CBX2021 had a positive impact on the gut microbiota as a whole.

LEfSe analysis was used to study the key bacterial species that drive variations within the microbial population. As shown in Figure 1E, a total of 33 different taxa were found in both groups (LDA score ≥ 3.0). In the CB piglets, seven genera, including *Faecalibacterium*, *Ligilactobacillus*, *Eubacterium_eligens_group*, and *Lachnospiraceae_NK4A136_group*, were significantly enriched and could serve as biomarker genera. Furthermore, CON piglets exhibited 14 characteristic genera, such as *unclassified_Muribaculaceae*, *Rikenellaceae_RC9_gut_group*, *Desulfovibrio*, and *Family_XIII_AD3011_group*.

### Supplementation with CBX 2021 altered the serum metabolome in weaned piglets

**Figure 2:**
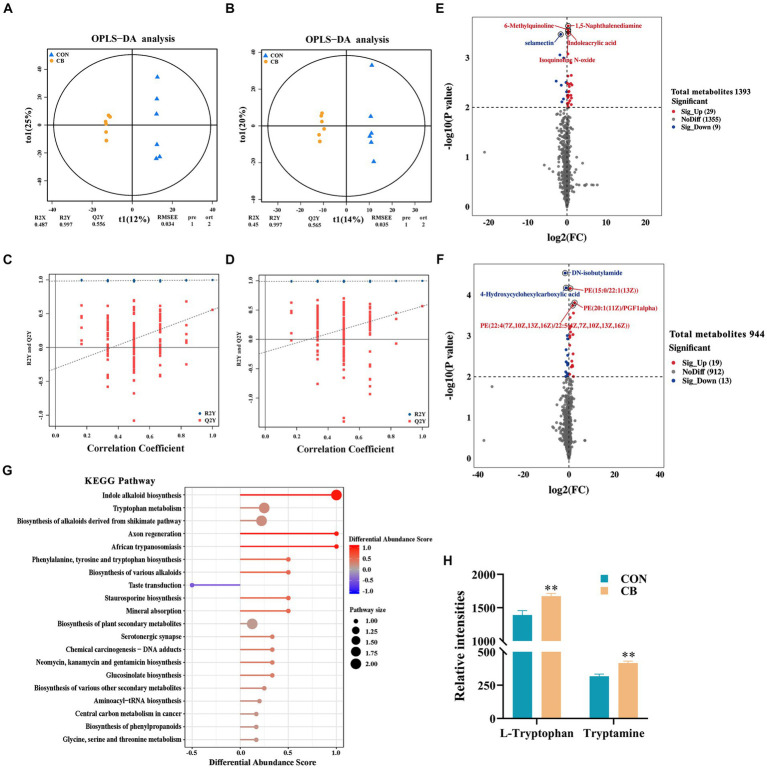
Effects of *Clostridium butyricum* CBX 2021 on serum metabolome in weaned piglets (*n* = 6). Orthogonal partial least squares discriminant analysis (OPLS-DA) score chart under positive **(A)** and negative **(B)** ion modes. Permutation test chart under positive **(C)** and negative **(D)** ion modes. Difference statistics volcano plot under positive **(E)** and negative **(F)** ion modes. **(G)** KEGG enrichment pathway diagram of different metabolites in two groups of piglets. **(H)** Histogram of the differential metabolites (L-tryptophan and tryptamine) that cause KEGG enrichment. Data are displayed as the mean ± SEM. ** Indicates *p* < 0.01 in comparison with the CON piglets.

LC-Q/TOF-MS technology was employed to examine the serum metabolome changes in weaned piglets treated with CBX 2021.The OPLS-DA score plots clearly showed clear separation between the two groups, suggesting that CBX 2021 significantly influenced the serum metabolite composition in piglets (Figures 2A,B). The high statistical values of R^2^Y (explanatory ability parameter) and *Q*^2^ (predictive ability parameter) in the score plots demonstrated that the models had good applicability and predictability. Furthermore, in the permutation tests of both ion modes, the intercepts of the Q^2^Y fitted regression lines with the vertical axis were below 0, confirming the reliability of the OPLS-DA model and its suitability for identifying differential metabolites between groups (Figures 2C,D).

A total of 70 differential metabolites were screened between the two groups of piglets, with 38 being positive ions and 32 being negative ions (Figures 2E,F and Supplementary Table S3). In the positive ion mode, the contents of 29 metabolites, such as 1,5-naphthalenediamine, 6-methylquinoline, indoleacrylic acid, and isoquinoline N-oxide, were significantly higher in the CB piglets than CON piglets, while the contents of 9 metabolites, such as selamectin, were significantly lower (*p* < 0.01). In the negative ion mode, the contents of 19 metabolites, such as PE(22:4(7Z,10Z,13Z,16Z)/22:5(4Z,7Z,10Z,13Z,16Z)), PE(20:1(11Z)/PGF1alpha), and PE(15:0/22:1(13Z)), showed a significant increase, while the contents of 13 metabolites, such as DN-isobutylamide and 4-hydroxycyclohexylcarboxylic acid, exhibited a significant decrease (*p* < 0.01).

Subsequently, an analysis of KEGG pathway enrichment was conducted on all differential metabolites found in the piglets from both groups. As shown in Figure 2G, CBX 2021 highly affected 5 pathways, including indole alkaloid biosynthesis, and tryptophan metabolism (*p* < 0.05). The significant increase in L-tryptophan and tryptamine mediated by CBX 2021 mainly contributed to the enrichment of these pathways (*p* < 0.05, Figure 2H). These outcomes show that CBX 2021 modulated tryptophan metabolism and biosynthesis of various alkaloids by altering the levels of metabolites, thus affecting the metabolic function of weaned piglets.

### Correlation analysis of microbiota, differential metabolites and blood biochemical indicators

**Figure 3:**
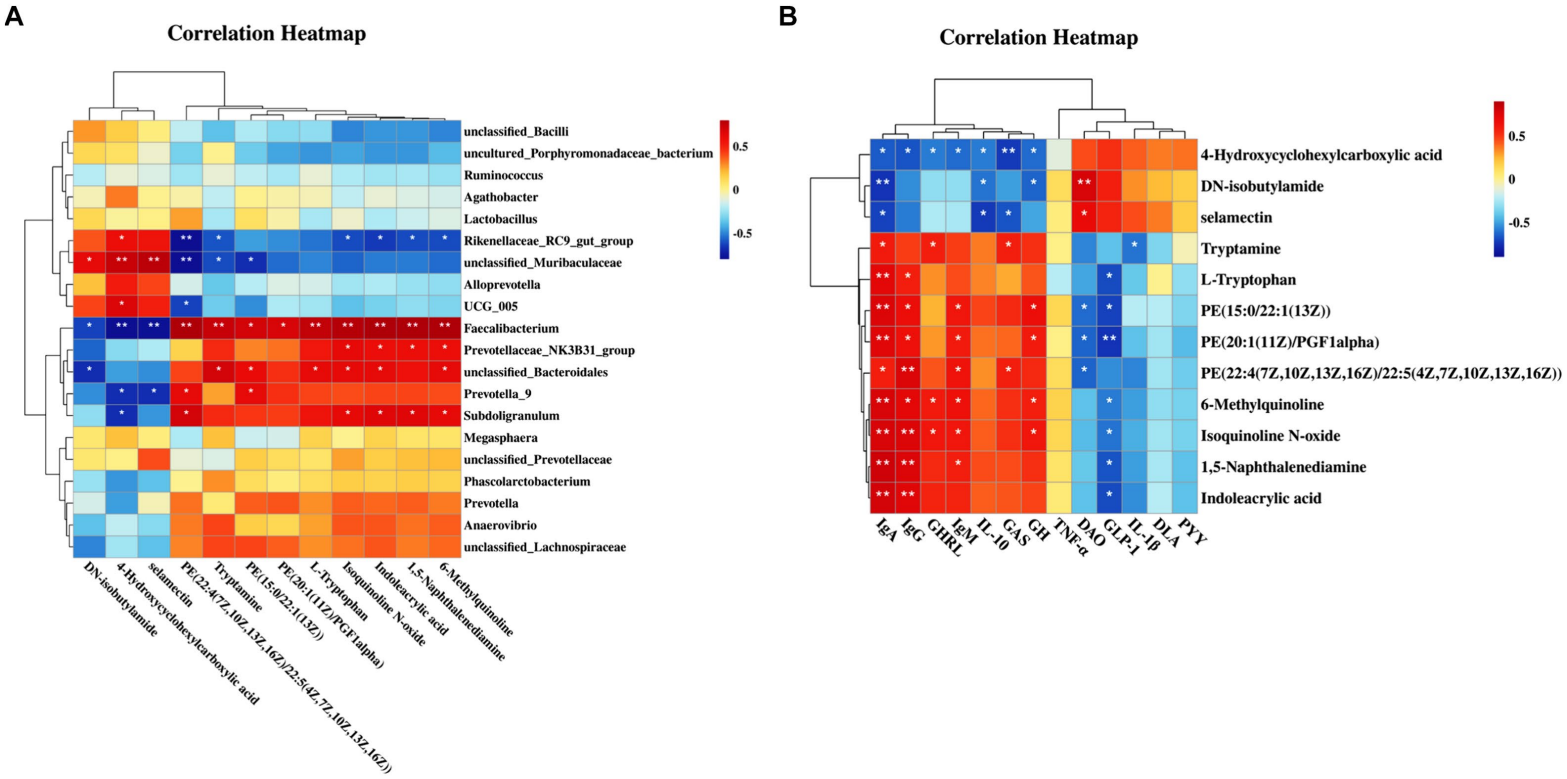
Correlation of microbiota, metabolites, and blood biochemical indicators (*n* = 6). **(A)** Heatmap of correlations between the top 20 bacterial genera and serum differential metabolites; The abscissa is the fecal bacteria with relative abundance of top 20; The ordinate is 12 serum differential metabolites. **(B)** Heatmap of correlations between serum differential metabolites and blood indicators; The abscissa is 12 serum differential metabolites; The ordinate is the blood biochemical indicators (immunoglobulin A/G/M, ghrelin, interleukin-10, growth hormone, gastrin, tumor necrosis factor-α, Glucagon-like peptide-1, diamine oxidase, interleukin-1β, peptide YY, D-lactic acid).

Metabolomics is considered an important tool for elucidating potential interactions between the microbiota and host phenotypes. Therefore, this study investigated the correlations between the top 20 bacterial genera and serum differential metabolites, along with the relationships between these metabolites and blood indicators. Figure 3A shows that in the CB piglets, upregulated genera (*Faecalibacterium*, *unclassified_Bacteroidales*, and *Subdoligranulum*) and downregulated genera (*unclassified_Muribaculaceae* and *Rikenellaceae_RC9_gut_group*) were highly correlated with differential metabolites. Specifically, *Faecalibacterium* showed positive correlations with upregulated metabolites in the CB piglets; these metabolites included 6-methylquinoline, indoleacrylic acid, isoquinoline N-oxide, L-tryptophan, and tryptamine (*p* < 0.05). *Faecalibacterium* exhibited negative correlations with downregulated metabolites in the CB piglets; these metabolites included selamectin, 4-hydroxycyclohexylcarboxylic acid, and DN-isobutylamide (*p* < 0.05). Interestingly, *unclassified_Bacteroidales* and *Subdoligranulum* showed similar trends to that of *Faecalibacterium*, while *unclassified_Muribaculaceae* and *Rikenellaceae_RC9_gut_group* showed opposite trends.

The correlation analysis results between differential metabolites and blood indicators are shown in Figure 3B. Serum immunoglobulins showed positive correlations with a majority of the upregulated metabolites in the CB piglets (*p* < 0.05), while they showed negative correlations with downregulated metabolites, especially 4-hydroxycyclohexylcarboxylic acid (*p* < 0.05). Regarding inflammatory factors, IL-1β was negatively correlated with tryptamine, and IL-10 was negatively correlated with selamectin, 4-hydroxycyclohexylcarboxylic acid, and DN-isobutylamide (*p* < 0.05). Notably, growth-related hormones, including GH, GHRL, and GAS, were consistent with the trend of immunoglobulin. Furthermore, in terms of indicators reflecting intestinal permeability, serum DAO activity showed positive correlations with the content of DN-isobutylamide and selamectin (*p* < 0.05), and it showed negative correlations with the content of PE(22:4(7Z,10Z,13Z,16Z)/22:5(4Z,7Z,10Z,13Z,16Z)), PE(20:1(11Z)/PGF1alpha), and PE(15:0/22:1(13Z)) (*p* < 0.05).

The aforementioned findings indicate that alteration in gut microbiota composition mediated by CBX 2021 intervention may positively influence hormone levels, immune responses and gut barrier function by regulating metabolic function, ultimately improving growth, development and overall health of piglets.

### Adhesion of CBX 2021 promoted the proliferation and reduced the inflammatory response in IPEC-J2 cells

**Figure 4:**
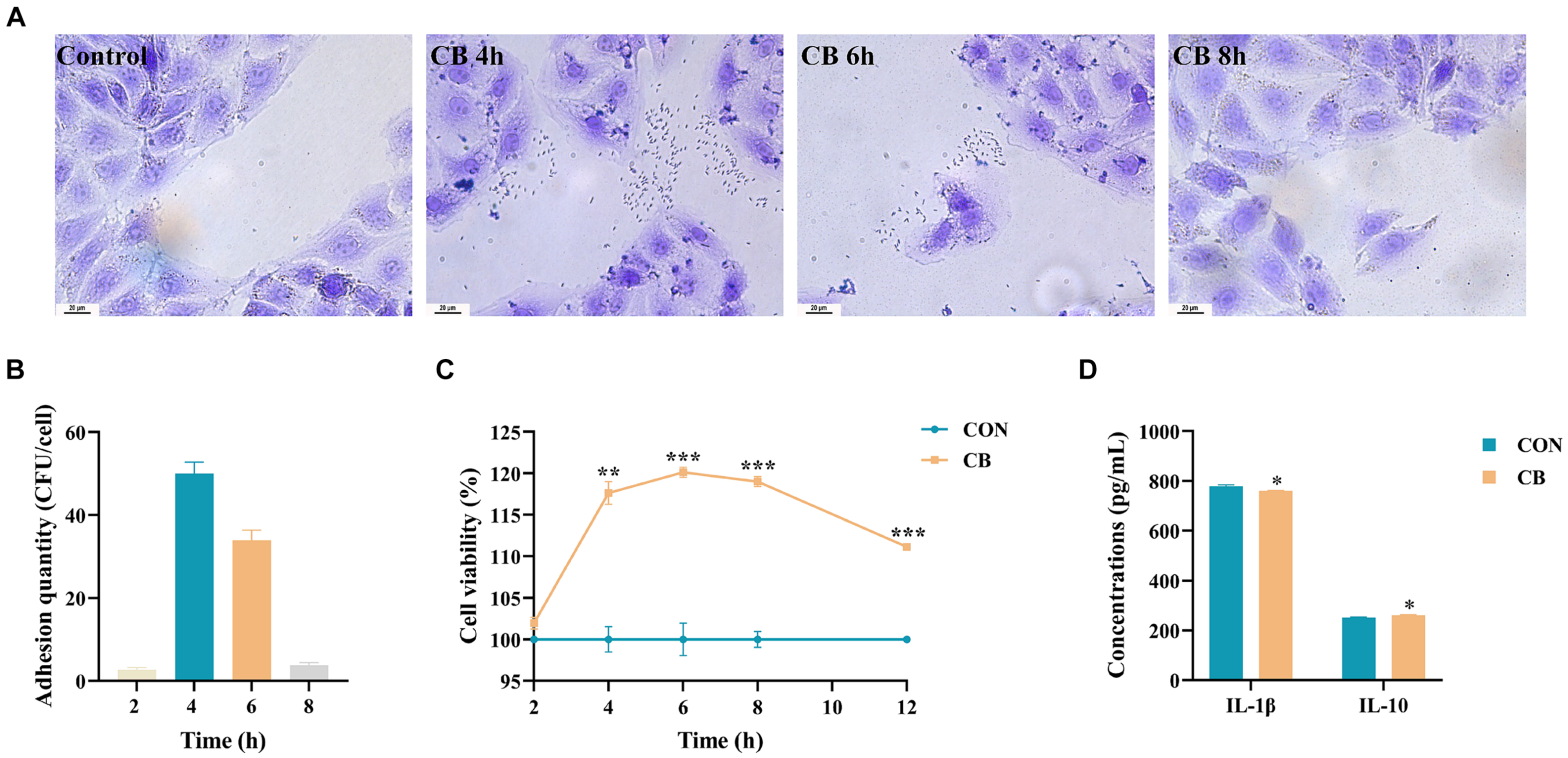
Effects of *Clostridium butyricum* CBX 2021 adhesion on the proliferation and inflammatory factors in IPEC-J2 cells (*n* = 25 or 3). **(A)** Gram staining microscopy of IPEC-J2 cells (scale bar = 20 μm). **(B)** Adhesion quantity of the CBX 2021 strain cocultured with IPEC-J2 cells at different time. **(C)** Line chart of cell proliferation activity. **(D)** Histogram showing the difference in inflammatory cytokine levels in the cell supernatant between two groups. Data are displayed as the mean ± SEM. *** Indicates *p* < 0.001, ** indicates *p* < 0.01, and * indicates *p* < 0.05 in comparison with the CON group.

Adhesion of strains to the surface of intestinal epithelial cells (IEC) is crucial for achieving their maximum probiotic effect (Kainulainen et al., 2015). Therefore, we assessed the ability of the CBX 2021 strain to adhere to IPEC-J2 cells in this study. The results, shown in Figures 4A,B, indicate that after 2 h of co-culture, there was almost no bacterial adhesion. After 4 h of co-culture, the average bacteria amount of adhesion was 50.04 ± 2.74 CFU/cell. After 6 h of co-culture, the average bacterial adhesion decreased to 33.92 ± 2.48 CFU/cell. When co-cultured for 8 h, there was almost no detectable bacterial adhesion. These findings indicate that the adhesion capability of CBX 2021 to IPEC-J2 cells is time-dependent, reaching its peak at approximately 4 h of co-culture.

The integrity of the intestinal barrier is dependent on the proliferation of IEC (Xie et al., 2019). Therefore, we investigated the impact of CBX 2021 adhesion on the proliferation activity of IPEC-J2 cells. The results in Figure 4C indicate that the strain adhesion did not significantly affect cell viability through 2 h of co-culture (*p* > 0.05). However, between 4 to 12 h of co-culture, CBX 2021 demonstrated a significant proliferative effect on IPEC-J2 cells (*p* < 0.01). As the co-culture time increased, cell viability gradually increased and reached its peak at 6 h, with a value of 120.14%. Subsequently, with a further increase in time, cell viability slightly declined, reaching 111.15% at 12 h. However, the CB group cells still demonstrated a significant proliferative effect on the cells at this time point (*p* < 0.001) compared to the CON group cells. The outcomes suggest that CBX 2021 significantly promoted IPEC-J2 cells proliferation, reaching peak activity after a 6-h co-culture. Therefore, a 6-h co-culture time was chosen for subsequent experiments.

To assess the potential anti-inflammatory properties of CBX 2021 in the intestine, we also analyzed the levels of inflammatory mediators in the cell supernatant. The data shows that CBX 2021 effectively decreased IL-1β levels, while enhancing IL-10 secretion in IPEC-J2 cells following 6 h of stimulation, (*p* < 0.05, Figure 4D). These findings are consistent with the *in vivo* experimental results, and thus suggesting that CBX 2021 can reduce the inflammatory response of IEC.

### Adhesion of CBX 2021 altered inflammation and immune regulatory genes in IPEC-J2 cells

**Figure 5:**
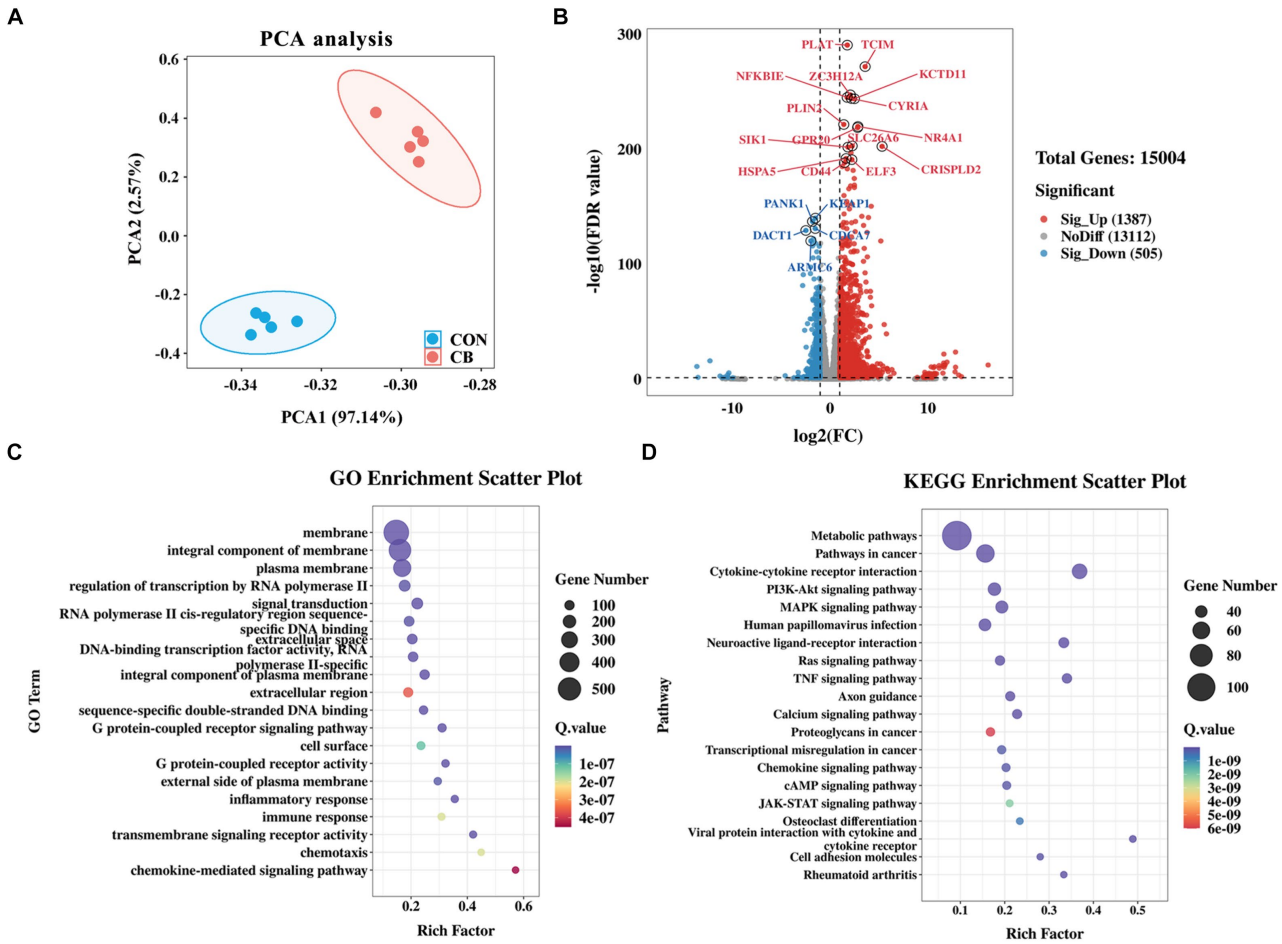
Effects of *Clostridium butyricum* CBX 2021 adhesion on the transcriptomics in IPEC-J2 cells (*n* = 5). **(A)** Principal component analysis (PCA) plot between the CON and CB RNA-seq samples. **(B)** The volcano plot displays differentially expressed genes (DEGs) from CON vs. CB. Bubble diagram of GO **(C)** and KEGG **(D)** enrichment analysis of differentially expressed genes.

To delve deeper into the molecular mechanisms responsible for reducing inflammation in IPEC-J2 cells through CBX 2021 adhesion, RNA-seq analysis was conducted to confirm changes in the relative gene expression. The PCA scatter plot indicates that the two groups of cells formed distinct clusters, suggesting significant differences in gene expression profiles (Figure 5A). A total of 1892 DEGs were distinguished between the CON and CB groups. Among them, 1,387 genes, including TCIM, ZC3H12A, NFKBIE, and GPR20, showed a marked increase in the CB group compared to the CON group, while 505 genes, including KEAP1, PANK1, and CDCA7, were significantly downregulated (Figure 5B). The heatmap was generated using the top 100 genes with the smallest adjust *p*-value (Supplementary Figure S1). It is obvious that cell samples within the same group have high repeatability in gene expression. Notably, the TCIM and ZC3H12A genes were mapped to GO database and involved in various immune-related pathways. For instance, the TCIM gene was implicated in negative regulation of the Notch signaling pathway. The ZC3H12A gene is involved in activating immune response signals and negatively regulating the production of IL-1β, IL-6, and TNF. The GPR20 gene is involved in the G protein-coupled receptor (GPCR) signaling pathway (Supplementary Table S4).

Subsequently, enrichment analyses were carried out for all DEGs using GO and KEGG databases. The findings showed that numerous DEGs were significantly enriched in GPCR signaling pathway, inflammatory and immune response, which are related to cellular health and stress adaptation (Figure 5C). The KEGG pathway analysis revealed multiple immune and inflammatory pathways, including cytokine-cytokine receptor interaction (52 DEGs), PI3K-Akt signaling pathway (44 DEGs), JAK–STAT signaling pathway (23 DEGs), were altered by the adhesion of CBX 2021 (Figure 5D). By screening for these DEGs, we found that IL10RA and IL22RA1, which are anti-inflammatory cytokine receptors, were significantly upregulated in the CB-treated cells. In contrast, the pro-inflammatory gene IL17D was significantly downregulated in the cells.

**Figure 6:**
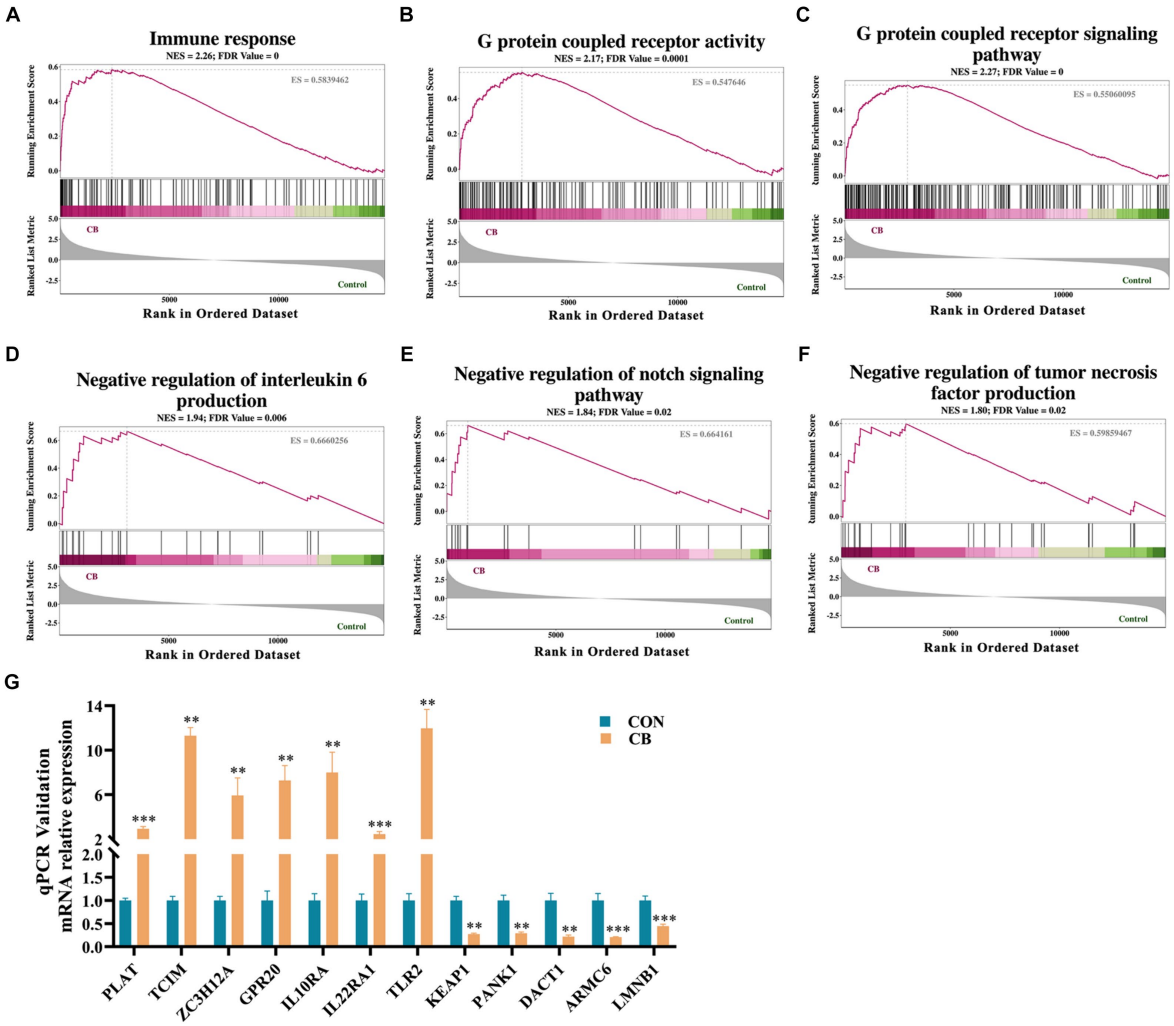
Gene set enrichment analysis (GSEA) pathway analysis and qRT-PCR validation **(A–F)** GSEA signal pathway analysis. **(G)** Histograms of target gene expression levels showed by qRT-PCR. Data are displayed as the mean ± SEM. *** Indicates *p* < 0.001 and ** indicates *p* < 0.01 in comparison with the CON group.

To further reveal the details of changes in cellular immunity and inflammatory response, GESA was conducted using the Go database on all gene sets of the CON and CB groups. As shown in Figures 6A–F, adhesion of CBX 2021 significantly enhanced immune response (ES = 0.58, NES = 2.26), GPCR activity (ES = 0.55, NES = 2.17), and GPCR signaling pathway (ES = 0.55, NES = 2.27) in the cells. In addition, CBX 2021 was able to improve the negative regulation for the Notch signaling pathway (ES = 0.66, NES = 1.85), the negative regulation of the production of IL-6 (ES = 0.67, NES = 1.94) and the negative regulation of tumor necrosis factor (ES = 0.60, NES = 1.80). The changes in these gene sets supported the fact that CBX 2021 decreased pro-inflammatory cytokines like IL-1β in IPEC-J2 cells. It is obvious that this result interprets the molecular pathway for CBX adhesion to reduce cellular inflammation at the gene level.

Next, the transcriptomic result was validated by using qRT-PCR method. Twelve genes (*PLAT*, *TCIM*, *ZC3H12A*, *GPR20*, *IL10RA*, *IL22RA1*, *TLR2*, *KEAP1*, *PANK1*, *DACT1*, *ARMC6*, and *LMNB1*) were subjected to qRT-PCR analysis. The findings indicated a high degree of concordance between the qRT-PCR data and transcriptomics results and thus confirming the reliability of the RNA sequencing (Figure 6G).

## Discussion

Weaning is often associated with impaired immune and metabolic function, compromised small intestinal structure, and dysbacteriosis, leading to postweaning diarrhea and growth retardation. In the context of feed antibiotic bans, CB, an important member of the animal gut symbiotic bacteria, helps to alleviate weaning stress and improve overall health (Chen L. et al., 2018). In this study, we demonstrated that porcine-derived CBX 2021, which has independent intellectual property rights, had the ability to enhance growth rate and lower diarrhea frequency in piglets. This is achieved by modulating gut microbiota structure, optimizing metabolic functions, reducing intestinal permeability, enhancing intestinal and serum immune functions, and promoting IPEC-J2 cell proliferation.

In our study, the experiment demonstrated that supplementing CBX 2021 improved piglets’ growth performance. This aligns with earlier studies suggesting that CB can improve the well-being of weaned piglets, Ira rabbits and calves (Li et al., 2019; Ye et al., 2022; Wu et al., 2023). Clearly, these reports also reflect that improving intestinal health is effective in alleviating the growth stress in animals caused by weaning. In addition, the enhancement in growth performance may be attributed not solely to the amelioration in peripheral hormone levels, but also to the reduction in intestinal permeability, which contributes to improved digestion and absorption of nutrients within the intestine (Guilloteau et al., 2006; Goes et al., 2022; Lee et al., 2022). Post-weaning diarrhea, primarily resulting from an imbalance in intestinal microbiota and the rapid proliferation of pathogens, is the chief contributor to growth retardation in animals (Rhouma et al., 2017). Research findings have indicated a strong correlation between reduction of diarrhea and the subsequent improvement in growth performance, and factors such as immune, intestinal barrier integrity, gut microbiota, and bacterial metabolism (Lu et al., 2020; Zhang R. Q. et al., 2021; Yu et al., 2022; Chen et al., 2023). In this research, CBX 2021 administration effectively reduced postweaning diarrhea in piglets, confirming the efficacy of *Clostridium butyricum* in improving the gut microbiota and restoring gut health. Thus, the overall health and development of the CB piglets was better than that of the CON piglets.

CBX 2021 effectively enhanced immunity and reduced inflammation in piglets. Specifically, on the one hand, the increased serum immunoglobulin concentration of CB piglets suggests that the ability to resist pathogen infection may be stronger. This observation aligns with previous research (Zhang et al., 2014; Han et al., 2020; Wang et al., 2021). A previous study showed that CB can promote IgA secretion by inducing an increase in IL-17-producing CD4+ T cells (Hagihara et al., 2021). However, we found that the improvement in immunity may be related to the upregulated metabolites mediated by CBX 2021, including phosphatidylethanolamine (PE), L-tryptophan, and bacterial tryptophan metabolites. PE supplementation has been reported to effectively restore impaired humoral immune function (Fu et al., 2021). L-tryptophan and its derivatives have demonstrated roles in modulating host immunity and maintaining intestinal balance (Gao et al., 2018). Therefore, CBX 2021 may promote immunoglobulin secretion by positively regulating the metabolite profile. On the other hand, CBX 2021 significantly reduced IL-1β levels and promoted IL-10 production in piglets mirroring the results reported by Li et al. (2018). IL-1β is a typical inflammatory mediator, and its overexpression can damage the animal intestine (Kruse et al., 2008). Conversely, IL-10 serves as an anti-inflammatory regulator that not only inhibits the overexpression of proinflammatory factors, but also regulates intestinal homeostasis during immune defense processes (Gao et al., 2012; Zigmond et al., 2014; Xu S. Q. et al., 2020). Generally speaking, CB primarily regulates the secretion of inflammatory cytokines through Toll-like receptor (TLR) pathways, such as TLR4/NF-κB pathway and TLR2/MyD88-independent pathway (Hayashi et al., 2013; Wang et al., 2022).Then, we further uncovered the potential molecular pathways that contribute to anti-inflammatory properties of CBX 2021 through transcriptomics analysis. Interestingly, GSEA intuitively showed that CBX 2021 enhanced GPCR activity and its signaling pathway, while downregulating the Notch signaling pathway and the production of proinflammatory factors (IL-6 and TNF-α).

Research indicates that elevated levels of Notch signaling within the intestine can suppress goblet cell differentiation, causing a rise in intestinal permeability and invasion of exogenous harmful substances, thereby inducing inflammation (Xie et al., 2020). Probiotics, such as *Bacillus subtilis* RZ001 and *Lactobacillus acidophilus* LA85, can repair the intestinal barrier and alleviate inflammation through inhibiting the Notch signaling pathway (Li et al., 2020; Xue et al., 2022). These studies were strongly supported in the current research. Notably, GPCRs have the potential to regulate immune activity and anti-inflammatory properties. For example, the researcher discovered that activation of GPR39 led to an increase in IL-10 release, which ultimately reduced inflammation in macrophages (Muneoka et al., 2018). Furthermore, activation of GPR109A has been shown to inhibit downstream Akt and NF-κB p65 phosphorylation in macrophages, thereby maintaining the barrier integrity and suppressing colonic inflammation (Chen G. X. et al., 2018). Therefore, it is speculated that CBX 2021 may reduce inflammation by activating the GPCR pathway and downregulating the Notch signaling pathway, thereby effectively reducing intestinal diseases such as diarrhea. Moreover, we hypothesized that after CBX 2021 adheres to cells, it may induce changes in cellular inflammation levels and gene expression profiles through its cell wall component, lipoteichoic acid (LTA). Research has found that *Clostridium butyricum* LTA can affect intracellular inflammatory responses by mediating NF-κB and ERK pathways (Wang H. et al., 2016). However, this is merely a preliminary guess, and we plan to further explore this mechanism through additional experiments in future research.

Weaning-induced inflammation often disrupts intestinal barrier function, leading to negative impacts on growth, development and general well-being (Stewart et al., 2017). In the present research, CBX 2021 reduced intestinal permeability and ameliorated weaning-induced intestinal barrier damage by reducing DLA and DAO levels. This is similar to reports in broiler chickens afflicted with *Escherichia coli* K88 and rats experiencing pancreatitis (Zhang et al., 2016; Zhao et al., 2020). Reducing intestinal permeability has proven to enhance piglet growth performance and reduce the incidence of diarrhea (Huang et al., 2015). And the recovery of intestinal barrier function is related to the cytokine levels and the GPCR pathway activated by CB (Lee, 2015; Ariyoshi et al., 2021). With respect to cytokines, proinflammatory cytokines can impair intestinal tight junctions and increase permeability, whereas anti-inflammatory cytokines protect and maintain intestinal barrier integrity (Lee, 2015). CBX 2021 did indeed induce positive changes in cytokine levels. In addition, we found that the improvement in intestinal permeability may be related to CBX 2021-mediated increases in a number of PE metabolites. PE is a vital constituent of cell membranes and has been demonstrated to play a significant role in repairing damaged intestinal cells damaged intestinal cells (Sawai et al., 2001).

Based on this, we further explained the potential mechanism of CBX2021 at the cellular level. The outcomes indicated that the adhesion of the CBX 2021 strain significantly promoted the proliferation of IPEC-J2 cells. Intestinal cell proliferation is a major driving force for intestinal growth and development, which is beneficial for repairing damaged intestinal barriers and preventing intestinal infections (Wang J. L. et al., 2019). Previous research has shown that stimulating PI3K/Akt and GPR84 pathway can induce the multiplication of mammary epithelial cells and promote mammary gland development (Meng et al., 2017). In our study, CBX 2021 significantly enhanced GPCR activity and its signaling pathway, as well as regulated the PI3K/Akt signaling pathway. Therefore, CBX 2021 might promote intestinal cell proliferation by modulating the GPCR and PI3K/Akt signaling pathways, thereby exerting probiotic effects on piglet intestinal structure.

Undoubtedly, CBX2021 has a regulatory effect on piglets by inducing changes in their gut microbiota. In our research, CBX2021 optimized the composition and structure of the fecal microorganism in weaned piglets. Notably, CBX 2021 significantly decreased the number of proinflammatory bacteria, including *unclassified_Muribaculaceae*, *Rikenellaceae_RC9_gut_group*, and *Family_XIII_AD3011_group*. Studies have indicated that the lower the abundance of these bacteria, the less the content of pro-inflammatory factors including IL-1β, IL-5 and IL-6 (Cai et al., 2021; Zhang L. et al., 2021; Zhang et al., 2022). The decrease in IL-1β content observed in this study is consistent with these findings. Additionally, CBX 2021 markedly decreased the abundance of *Desulfovibrio*. The bacterium secretes toxic metabolites that harm IEC and immune function (Verstreken et al., 2012; Kushkevych et al., 2018). It is speculated that the decrease in these bacteria may be one of the reasons for the decrease in the incidence of diarrhea in piglets.

In addition, CBX 2021 enriched many functional bacteria that produce butyric acid, including *Faecalibacterium*, *[Eubacterium]_eligens_group*, and *Lachnospiraceae_NK4A136_group*. These bacteria serve as anti-inflammatory regulators and enhance intestinal barrier function (Dou et al., 2020; Xu J. H. et al., 2020; Tang S. S. et al., 2022). For instance, *Faecalibacterium* exerts anti-inflammatory actions by suppressing histone deacetylase activity through producing butyric acid (Zhou et al., 2018). The *Lachnospiraceae_NK4A136_group* has been shown to promote the expression of the tight junction protein claudin-1, which is negatively correlated with intestinal permeability and serum LPS levels (Ma et al., 2020). Additionally, CBX 2021 promoted the growth of helpful bacteria, including *Prevotella_9* and *Ligilactobacillus*. Notably, *Prevotella_9* has demonstrated a positive relationship with weight, ADG, and ADFI, and a negative relationship with diarrhea incidence (Hung et al., 2019; Yang et al., 2021). Therefore, CBX 2021 may have beneficial effects on weaned piglets by modulating the gut microbiota.

Alterations in the gut microbiome undoubtedly result in changes in metabolic function and levels of metabolites (Ariyoshi et al., 2021). These metabolites act as messengers of the intestinal microbiota, directly regulating various physiological processes in the host. CBX 2021 significantly enhanced the biosynthesis of various alkaloids, including indole alkaloids, and tryptophan metabolism. Studies have demonstrated that indole alkaloids exhibit a range of advantageous effects, such as anti-inflammatory, anti-pathogenic bacteria, and antioxidant properties (Song et al., 2018; Hu et al., 2021). Therefore, it is reasonable to believe that the reduction in proinflammatory mediators and harmful bacteria in the intestines of weaned piglets may be partly associated with these effects. Tryptophan and its derivatives, including kynurenine, indole, indoleacrylic acid, and tryptamine, play important roles in inflammatory responses (Kim et al., 2010; Bhattarai et al., 2020; Liu et al., 2022; Duan et al., 2023). Indoleacrylic acid, for example, can promote IL-10 production and enhance intestinal epithelial barrier function, resulting in anti-inflammatory effects (Wlodarska et al., 2017). In addition to regulating inflammation, tryptophan metabolism is also important for maintaining immune homeostasis, protecting intestinal integrity, and promoting animal growth (Yao et al., 2011; Liang et al., 2018; Seo and Kwon, 2023). It is worth noting that we found that the levels of L-tryptophan and tryptamine were positively correlated with the abundance of *Faecalibacterium* and *unclassified_Bacteroidales*. Therefore, CBX 2021 may boost tryptophan metabolism through the increased proliferation of these bacteria. This can positively regulate inflammation, immune function, and intestinal barrier function, ultimately promoting healthy growth of piglets.

## Conclusion

In conclusion, exogenous administration of CBX 2021 effectively reduced postweaning diarrhea and accelerated growth of piglets through enhancing immune function and intestinal barrier integrity. The mechanism is mainly related to the improvement of intestinal health by CBX 2021. CBX 2021 not only optimized gut microbiota structure and improved bacterial metabolic functions in piglets, and also directly promoted epithelial cell proliferation and reduced inflammation by altering cellular gene expression. Thus, the current research reveals the probiotic mechanism of *Clostridium butyricum* and establishes a theoretical foundation for the potential application of CBX 2021.

## Data availability statement

The raw 16S rRNA sequencing data and transcriptome sequencing data presented in the study are deposited in the NCBI Sequence Read Archive (SRA), accession number PRJNA947735 and PRJNA948058.

## Ethics statement

The animal study was approved by Ethics Committee of National Center of Technology Innovation for Pigs. The study was conducted in accordance with the local legislation and institutional requirements.

## Author contributions

XL: Writing – review & editing, Writing – original draft, Visualization, Validation, Supervision, Methodology, Investigation, Formal analysis, Data curation, Conceptualization. XQ: Writing – review & editing, Validation, Methodology, Investigation, Funding acquisition, Formal analysis, Data curation. YY: Writing – review & editing, Validation, Methodology, Investigation, Formal analysis, Data curation. JW: Writing – review & editing, Validation, Methodology, Investigation, Formal analysis, Data curation. QW: Writing – review & editing, Validation, Methodology, Investigation, Funding acquisition. JL: Writing – review & editing, Validation, Methodology, Investigation. JH: Writing – review & editing, Validation, Investigation, Conceptualization. FY: Writing – review & editing, Validation, Investigation, Conceptualization. ZL: Writing – review & editing, Writing – original draft, Validation, Supervision, Investigation, Funding acquisition, Conceptualization. RQ: Writing – review & editing, Writing – original draft, Validation, Supervision, Resources, Project administration, Methodology, Investigation, Funding acquisition, Conceptualization.

## Footnotes

^1^ https://www.biocloud.net

## Supplementary material

SUPPLEMENTARY FIGURE S1 Cluster analysis of the top 100 DEGs with the smallest *P*-value.
