# Supplementary figures and images for: Uncovering the mechanism of Clostridium butyricum CBX 2021 to improve pig health based on in vivo and in vitro studies
Source: Front Microbiol. 2024 Jun 14;15:1394332. doi: 10.3389/fmicb.2024.1394332 (PMC11211278; doi:10.3389/fmicb.2024.1394332)

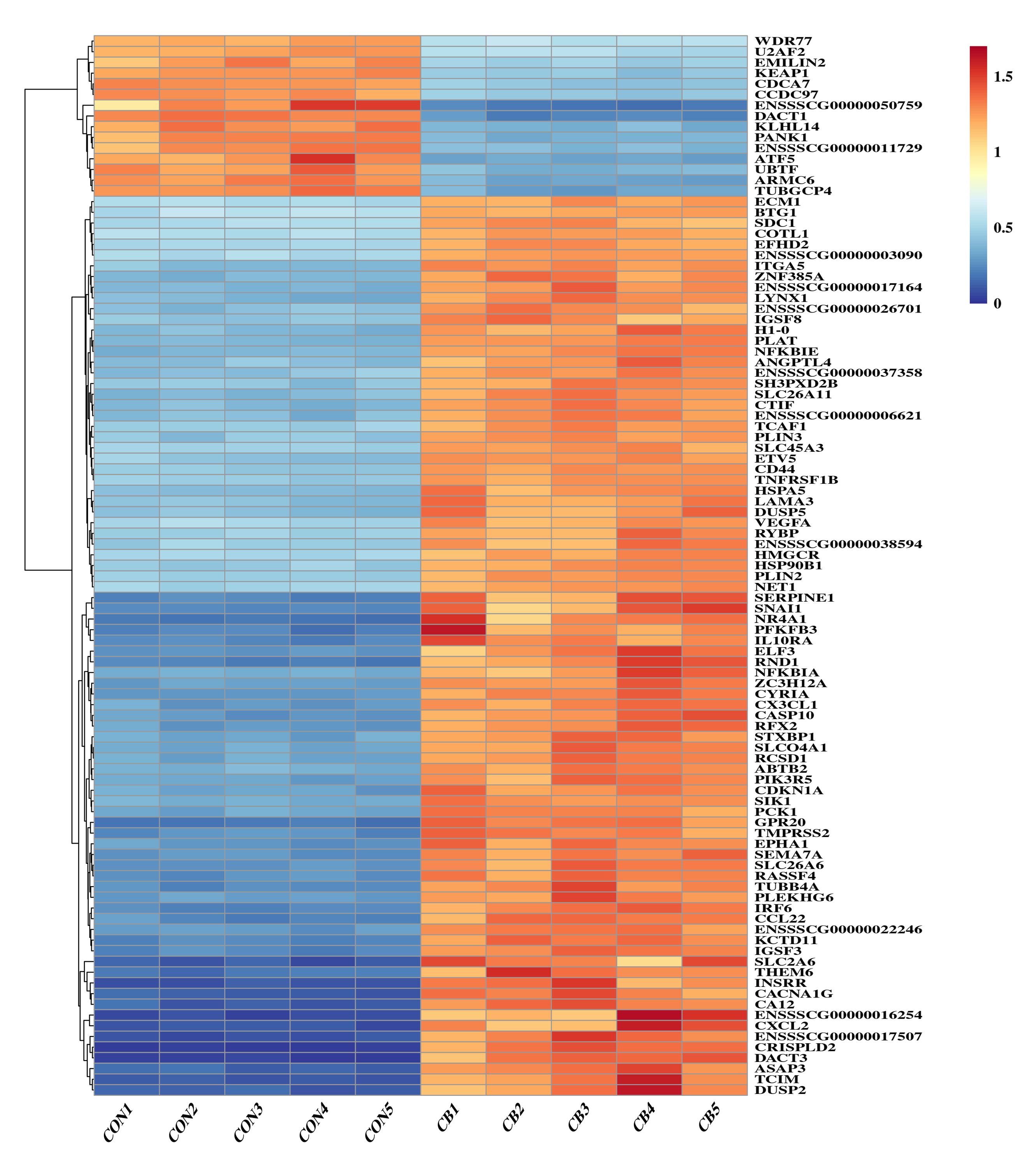

Supplement: SUPPLEMENTARY FIGURE S1 — Cluster analysis of the top 100 DEGs with the smallest P-value. [file Image_1.TIF]
